# Residential exposure to air pollution and access to neighborhood greenspace in relation to hair cortisol concentrations during the second and third trimester of pregnancy

**DOI:** 10.1186/s12940-021-00697-z

**Published:** 2021-02-11

**Authors:** Veerle Josefa Verheyen, Sylvie Remy, Nathalie Lambrechts, Eva Govarts, Ann Colles, Lien Poelmans, Els Verachtert, Wouter Lefebvre, Pieter Monsieurs, Charlotte Vanpoucke, Flemming Nielsen, Lena Van den Eeden, Yves Jacquemyn, Greet Schoeters

**Affiliations:** 1 Flemish Institute for Technological Research (VITO), Mol, Belgium; 2 Department of Biomedical Sciences, University of Antwerp, Antwerp, Belgium; 3 Belgian Interregional Environment Agency, Brussels, Belgium; 4 The Department of Public Health, University of Southern Denmark, Odense, Denmark; 5 Department of Obstetrics and Gynecology, Antwerp University Hospital, Antwerp, Belgium; 6 People and Health, Thomas More University College, Lier, Belgium; 7 Global Health Institute, Faculty of Medicine, University of Antwerp, Antwerp, Belgium; 8 Antwerp Surgical Training, Anatomy and Research Centre, University of Antwerp, Antwerp, Belgium

**Keywords:** Air pollution, Proximity to major roads, Neighborhood greenspace, Pregnancy, Longer-term biological stress, Hair cortisol concentrations

## Abstract

**Background:**

Exposure to air pollution during pregnancy has been associated with adverse pregnancy outcomes in studies worldwide, other studies have described beneficial effects of residential greenspace on pregnancy outcomes. The biological mechanisms that underlie these associations are incompletely understood. A biological stress response, which implies release of cortisol, may underlie associations of air pollution exposure and access to neighborhood greenspaces with health.

**Methods:**

We explored residential exposure to air pollution and residential access to neighborhood greenspaces in relation to hair cortisol concentrations of participants in a prospective pregnancy cohort study in Flanders, Belgium. Hair samples were collected at the end of the second pregnancy trimester (*n* = 133) and shortly after delivery (*n* = 81). Cortisol concentrations were measured in 3-cm scalp-near hair sections, to reflect second and third pregnancy trimester cortisol secretion. We estimated long-term (3 months before sampling) residential exposure to fine particulate matter (PM_2.5_), nitrogen dioxide (NO_2_) and black carbon (BC), assessed residential distance to major roads and residential access to neighborhood greenspaces (NHGS). Associations between residential exposures and hair cortisol concentrations were studied using linear regression models while adjusting for season of sampling.

**Results:**

Three-month mean residential NO_2_ and BC concentrations were positively associated with third pregnancy trimester hair cortisol concentrations (*p* = 0.008 and *p* = 0.017). Access to a large NHGS (10 ha or more within 800 m from residence) was negatively associated with third trimester hair cortisol concentrations (*p* = 0.019). Access to a large NHGS significantly moderated the association between residential proximity to major roads and second trimester hair cortisol concentrations (*p* = 0.021). Residential distance to major roads was negatively associated with second trimester hair cortisol concentrations of participants without access to a large NHGS (*p* = 0.003). The association was not significant for participants with access to a large NHGS. The moderation tended towards significance in the third pregnancy trimester (*p* < 0.10).

**Conclusions:**

Our findings suggest a positive association between long-term residential exposure to air pollution and biological stress during pregnancy, residential access to neighborhood greenspaces may moderate the association. Further research is needed to confirm our results.

**Trial registration:**

The IPANEMA study is registered under number NCT02592005 at clinicaltrials.gov.

**Supplementary Information:**

The online version contains supplementary material available at 10.1186/s12940-021-00697-z.

## Background

In the past decade, epidemiological studies throughout the world have linked maternal exposure to road traffic and air pollution to adverse pregnancy outcomes such as low birth weight, preterm birth and intrauterine growth restriction [1–3]. These adverse birth outcomes not only increase perinatal morbidity and mortality, but also increase susceptibility to obesity, diabetes and cardiovascular diseases later in life [4, 5]. Exposure to air pollution may also affect maternal health, ambient air pollution has been linked to hypertensive pregnancy disorders (gestational hypertension, pre-eclampsia) and gestational diabetes mellitus [6–8]. Both conditions amplify young women’s risk of developing cardiovascular diseases later in life [9]. The adverse impact of maternal exposure to air pollution on birth outcomes is of major public health importance, considering the ubiquitous nature of air pollution in urban settings [10]. The biological pathways that underlie associations between maternal exposure to air pollution and adverse pregnancy outcomes however, remain incompletely understood. Recent experimental animal research has shown that a neuroendocrine stress response is among the early biological responses triggered by exposure to fine particulate matter with an aerodynamic diameter ≤ 2.5 μm (PM_2.5_) and exposure to nitrogen dioxide (NO_2_) [11]. The biological stress response includes activation of the hypothalamic-pituitary-adrenal (HPA) axis and release of glucocorticoid stress hormones, with the glucocorticoid cortisol as its main downstream effector in humans [12]. The relevance of these experimental observations to humans has been confirmed in a few recent studies [13, 14]. To date, most human studies have assessed short-term variations in cortisol levels in relation to air pollution exposure, using blood and saliva as a matrix. Longer-term cortisol concentrations are difficult to evaluate using blood and saliva, due to circadian variations in cortisol secretion and the need for multiple sampling [15]. Repeated sampling increases discomfort for study participants. Hair however, is a suitable matrix for the assessment of longer-term cortisol concentrations [16]. As cortisol is incorporated into growing hair, hair cortisol concentrations (HCC) retrospectively reflect cortisol secretion over a period of several months [17]. The validity of HCC as an index of long-term systemic cortisol concentrations has been demonstrated in direct and indirect validation studies [18]. Strong positive associations between HCC and mean salivary cortisol levels, obtained through repeated sampling, were first established in rhesus macaques and later confirmed in human studies, including a study in pregnant women [18, 19]. In experimental animal studies, repeated stimulation of cortisol secretion by administration of HPA-axis hormones (adrenocorticotropic hormone, corticotropin-releasing hormone), was associated with an increased accumulation of cortisol in hair [20]. Research has also associated HCC with conditions that are known to be related to altered adrenocortical function, such as Cushing’s syndrome, Addison’s disease and cardiovascular diseases [21]. Importantly, chronic activation of the maternal HPA axis during pregnancy has been associated with gestational hypertensive disorders, intrauterine growth restriction and developmental programming of disease susceptibility [22, 23]. With regard to birth outcomes, a significant negative association between maternal HCC in the 2nd pregnancy trimester and gestational age at delivery has been reported [24].

Interestingly, a growing body of research suggests a beneficial relationship between residential access to greenspaces (community gardens, urban parks, forests) and pregnancy outcomes for both mothers and babies [25]. Physiological stress recovery has been suggested as a potential biological pathway, linking residential access to neighborhood greenspace to health [26, 27]. A relationship between the use of urban neighborhood greenspace and decreased hair cortisol concentrations has previously been demonstrated in an adult population in Berlin, Germany [28]. According to the World Bank, 55% of the world’s population lived in cities in 2019, this trend is expected to continue in the coming decades [29]. Given the ubiquitous nature of air pollution in urban settings, residential access to neighborhood greenspaces may play an important role in protecting and promoting urban health [30]. The adverse effects of air pollution may, to some degree, be moderated by the beneficial effects of residential access to greenspace [31].

To our knowledge, no research is available on residential air pollution exposure or access to neighborhood greenspace in relation to longer-term biological stress during pregnancy. Accordingly, we aimed to explore 1) associations of residential exposure to air pollution and proximity to major roads with maternal hair cortisol concentrations during the second and third trimester of pregnancy and 2) associations of residential access to neighborhood greenspace with maternal hair cortisol concentrations during the second and third trimester of pregnancy. In addition, we aimed to explore whether residential access to a neighborhood greenspace moderated associations of residential exposure to air pollution and proximity to major roads with maternal biological stress.

## Methods

### Study population and design

This study was conducted in the framework of IPANEMA (Impact of Particulate Matter on Mothers and Babies in Antwerp), a prospective pregnancy cohort study of the Antwerp University Hospital (UZA) in collaboration with the Flemish Institute for Technological Research (VITO) and the University of Antwerp (UA). Pregnant women were recruited between April 2015 and January 2018 at the UZA prenatal clinic by a midwife or obstetrician at a gestational age of 12 to 14 weeks. The inclusion criteria were: a singleton pregnancy; the ability to fill out extensive Dutch questionnaires; delivery planned in the Antwerp University Hospital. All participating mothers gave written informed consent. The study protocol was approved by the ethical committee of the University of Antwerp (14/40/411) and registered under number NCT02592005 at clinicaltrials.gov. Health-related information on mothers and babies was extracted from the hospital records and questionnaires that participants completed at enrolment, during pregnancy and after delivery. These questionnaires provided detailed information on participants’ socio-demographic and lifestyle characteristics. A detailed protocol of the IPANEMA study can be found elsewhere [32].

### Residential exposure assessment

Assessment of all residential exposure variables was based on the participants’ geocoded home address. Geographical Information System (GIS) analyses were carried out using ESRI ArcGIS software version 10.4 (Environmental Systems Research Institute, Redlands, California, USA). The residential degree of urbanization was assessed according to the Eurostat definition that classifies local administrative units as cities, towns, suburbs or rural areas based on a combination of geographical contiguity and population density, applied to 1 km^2^ population grid cells [33]. We assessed residential exposure to fine particulate matter (PM_2.5_), nitrogen dioxide (NO_2_) and black carbon (BC), primary constituents of traffic-related air pollution. Residential exposure to PM_2.5_, NO_2_ and BC was modelled using a spatial temporal interpolation method. In Flanders, atmospheric pollutants are continuously measured by a network of automatic monitoring stations by the Flemish Environment Agency. The Belgian Interregional Environment Agency (IRCEL, Intergewestelijke Cel voor het Leefmilieu) uses these measurements together with information on land cover to interpolate the air pollutant concentrations on a 4 × 4 km^2^ resolution [34]. These background results are combined with a bi-gaussian dispersion model based on emissions from point sources and line sources, the Immission Frequency Distribution Model (IFDM). The combined RIO-IFDM model chain produces daily averaged pollutant concentrations in Belgium on a high resolution receptor grid [35]. We calculated mean air pollutant concentrations at the residential address over a 3-month period before sampling, similar to the period of cortisol accumulation in the hair samples, and over a 1-year period before sampling. Residential proximity to major roads is often used as a surrogate measure of long-term exposure to traffic-related air pollution [36]. We calculated the straight-line distance from each residence to the nearest major road. Major roads included international motorways (E-roads) and the network of large national and local roads of Belgium (N-roads).

Residential access to a neighborhood greenspace was based on the 2016 version of the land-use map of Flanders, which maps land cover types, i.e. natural vegetated land cover and urban greenery, in 10 × 10 m^2^ raster cells [37]. Green cells were clustered to assess the area and public accessibility of greenspace in the maternal residential surroundings. Access to a small neighborhood greenspace (NHGS) was defined as access to at least 0.2 ha (ha) of greenspace within a travel distance of 400 m (m) from residence, access to a large greenspace was defined as access to at least 10 ha of greenspace within a travel distance of 800 m from residence. In the large greenspace typology, small water bodies are included when surrounded by > 50% greenspace, agricultural land is included when surrounded by > 30% greenspace. More technical background information on the green typology indicators can be found elsewhere [38].

### Hair sample collection and cortisol measurement

At the end of second trimester of pregnancy in-hospital consultation and shortly after delivery, a strand of hair of at least 2 mm thick was bound together with a cotton thread and cut close to the scalp from the posterior vertex region of the head. This area of the scalp exhibits the lowest intra-individual variability in HCC [39]. Hair samples were stored in paper envelopes at room temperature until analysis. When protected from ultraviolet light, cortisol concentrations in hair samples remain stable at room temperature for several years [40]. Cortisol concentrations were determined from the 3 cm of hair closest to the scalp. Based on an average hair growth of 1 cm per month, this length represents cortisol secretion in a 3-month period, a trimester, prior to sampling [41]. The samples taken in this study are therefore indicative of hair cortisol concentrations during the second and third trimester of pregnancy. There is a wide consensus that the first 5–6 cm of hair nearest to a person’s scalp can reliably reflect HPA activity [42]. Analysis was performed at the Institute of Public Health, Department of Environmental Medicine of the University of Southern Denmark (SDU), using liquid chromatography and tandem mass spectrometry (LC-MS/MS) as described by Chen et al. [43], after minor modifications. Hair samples were washed with methanol and dried at room temperature. The 3 cm of hair closest to the scalp was cut into segments of 2–3 mm. A typical amount of hair weighed 20–30 mg. Aliquots of 100 μL 20 ng/mL isotope labeled cortisol (cortisol-D_4_) were added as internal standard, together with 0.9 mL methanol. Samples were incubated in the dark at 25 °C while whirl mixed at 2000 rpm for 5 days and subsequently centrifuged at 3000 *g* for 5 min. Twenty microliter of the supernatant was injected onto a High-Performance Liquid Chromatography (HPLC) column. HPLC was performed using an Accella 1250 pump (Thermo Scientific, San Jose, CA) and a PAL autosampler (CTC analytics, Zwingen, Switzerland). The analytical column was a Kinetex C18 column, 100 × 4.6 mm (2.6 μm) equipped with a 2 × 4 mm C18 SecurityGuard column (Phenomenex, Torrance, CA). Isocratic elution was performed with a mobile phase system consisting of methanol and 0.1 M formic acid (80:20) at a flow rate of 400 μL/min for 6 min. After the peaks were eluted, a wash procedure was performed before the next samples was injected onto the column. The triple quadrupole mass spectrometer utilized was a TSQ Vantage (Thermo Scientific, San Jose, CA). The calibration curve and calculation of the sample concentration were based on the area ratio of the analyte/isotope labeled internal standard. Quality control samples were included in each series of samples. The limit of quantification (LOQ) for cortisol was 1.0 pg/mg hair. The intra-day repeatability coefficient of variation was 8.7% and the inter-day reproducibility coefficient of variation was 9.5%.

### Potential covariates

The selection of potential covariates was based on existing literature [18, 44–48]. A meta-analysis of HCC research identified age and hair washing frequency as potential covariates of HCC [18]. Other studies have identified household socioeconomic status (SES), anthropometry, chronic diseases and the local use of glucocorticoids as potential covariates of HCC [44, 46, 47]. Studies on determinants of HCC in pregnant women suggested pre-pregnancy BMI, parity, season of sampling and gestational week at sampling as potential covariates of HCC during pregnancy [45, 48]. In this study, we had information on maternal age, parity, maternal socioeconomic status (SES) defined as the highest educational attainment of the mother and categorized as low/intermediate/high, pre-existing chronic diseases (diabetes, asthma, cardiovascular disease), pre-pregnancy body mass index (BMI), gestational week at sampling, season of sampling, smoking and alcohol consumption before pregnancy, systemic use of glucocorticoids and daily hair washing. We assessed maternal ethnic background as European/non-European country of birth since hair growth rate may be influenced by ethnicity [49]. We additionally assessed variables that may have a link with both residential environment and biological stress, i.e. neighborhood SES and residential exposure to noise. A systematic review in the World Health Organization (WHO) European Region showed that lower neighborhood SES is usually linked with higher levels of air pollutants [50]. Independent from higher levels of exposure, deprived mothers may have a higher vulnerability, leading to more pronounced adverse health effects of a given environmental exposure [51]. The Area Deprivation Index (ADI) is a yearly calculated indicator for neighborhood SES on a sub-municipality level in Flanders [52]. Deprivation is recorded by the Flemish Child and Family Government Agency (www.kindengezin.be). Selection criteria for deprivation are the family’s monthly income, the parents’ educational attainment, the children’s development, the parents’ employment situation, housing and health. If a family fulfils three or more criteria, it is considered to be underprivileged [53]. The index of year X (%), i.e. 2017, considers all children born in year X, X-1 and X-2 that live in deprived households in a given area in Flanders, divided by the total number of children born in the area during the same period. The ADI of the participants’ neighborhood was subsequently categorized into tertiles representing low, intermediate and high area deprivation across the range of ADI among all participants.

Residential proximity to major roads may also lead to elevated noise levels [54]. Residential noise exposure levels were assessed using the Flemish strategic noise map of 2016, which includes major road infrastructure as defined in the EU-guideline 2002/49/EG [55]. The strategic noise map expresses noise levels in L_den_, the average sound level over a 24 h period with a penalty of 5 dB added for evening hours and a penalty of 10 dB added for nighttime hours [56]. The WHO guideline for average noise exposure produced by road traffic is set at 53 dB (dB) L_den_, road traffic noise above this level has been associated with adverse health effects, including adverse birth outcomes [57]. Noise exposure was therefore evaluated binary as exposure to a noise level ≥ 53 dB L_den_.

### Statistical analysis

Statistical analysis was performed using SPSS Statistics (version 25; IBM, Armonk, NY, USA) and R (version 2018; R Foundation for Statistical Computing, Vienna, Austria). Descriptive statistics provide an overview of study population characteristics, residential exposure characteristics and geometric mean HCC concentration with 95% confidence interval. Air pollution variables were logarithmically transformed (ln-scale) because of skewed distributions, distance to major roads was logarithmically transformed to reflect the non-linear distance decay of traffic-related exposure to air pollutants [58].

Spearman rank correlations between residential exposures variables were assessed, since correlations of 0.9 or higher between exposure variables indicate strongly connected exposures that cannot be disentangled [59]. The outcome variable HCC was logarithmically transformed to obtain a normal distribution. For HCC below the LOQ of 1 pg/mg hair, a random imputation from a log-normal probability distribution was performed where the mean was allowed to depend on observed values for hair cortisone concentrations that were measured simultaneously with cortisol, since both glucocorticoids were highly correlated (*p* < 0.01, Pearson’s *r* = 0.711 for 2nd trimester cortisol and cortisone, *p* < 0.01, Pearson’s *r* = 0.758 for 3rd trimester cortisol and cortisone). Linear regression models were used to analyze associations between 3-month mean air pollutant concentrations (PM_2.5_, NO_2_, BC), distance to major roads and access to neighborhood greenspace as a predictor and 2nd and 3rd trimester HCC as an outcome. Given the limited number of study participants, we decided not to adjust for a set of a priori selected covariates. We assessed the significance of the aforementioned potential covariates in relation to HCC in this cohort by performing a univariate analysis of variance (ANOVA). We then specified two linear regression models. Model I was unadjusted, model II was adjusted for season of sampling, based on its significance as a covariate of HCC (*p* < 0.05) in the ANOVA. All assumptions of linear regression were checked. To quantify the associations of continuous exposure variables with HCC, the effect estimates with 95% confidence intervals (95% CI) are presented as the factor change in HCC for a factor increase in exposure from the 25th to the 75th percentile. The factor increase in exposure is calculated as the ratio of the 75th to the 25th percentile (p75/p25) of the exposure variable. To quantify the associations of access to neighborhood greenspace with HCC, the effect estimates (β) with 95% CI are presented for having access to neighborhood greenspace, compared to having no access to neighborhood greenspace.

Effect modification by access the neighborhood greenspace was assessed by adding the interaction term of exposure to air pollution or distance to major roads and access to neighborhood greenspace into the regression model. The significance of the interactions is reported (*p*-interaction). For significant interactions, the effect estimates (β) with their 95% CI are reported for participants with access to neighborhood greenspace and for participants without access to neighborhood greenspace. The level of significance for associations and interactions was set at *p* < 0.05.

We conducted several sensitivity analyses to evaluate our results. We tested 1-year mean air pollutant concentrations in relation to 2nd and 3rd trimester HCC to confirm the robustness of 3-month mean results. We additionally adjusted our models for frequency of hair washing, a factor that may influence HCC, independently of biological stress. We additionally adjusted the models for maternal age, pre-pregnancy BMI ≥ 25 (overweight and obese), educational attainment and ADI > the study mean of 16.4%, to evaluate potential residual confounding. As a final sensitivity analysis, associations between residential exposures and HCC were investigated while excluding participants that reported a non-European country of birth.

## Results

Hair samples for cortisol analysis were provided by 152 participants. Characteristics of the study population are described in Table 1. We excluded 3 participants due to inexplicable high HCC values (> 3 times the interquartile range above the third quartile). As a result, 149 pregnant women were included in this study, of which 133 women donated a sample at the end of the 2nd trimester (week 26 ± 1.6) and 81 women shortly after delivery (week 39 ± 1.6), 65 women donated a sample twice. Almost half of the 149 mothers (48%) was aged between 26 and 30 years, 61% of participants were primigravid. Most of the study participants were of European origin (75, 21% data missing), enjoyed higher education (57, 23% data missing) and were employed prior to their pregnancy (72, 23.5% data missing).

**Table 1:** Basic characteristics of the study participants

Characteristic	*n* (%)
Age	
≤ 25	17 (11.4)
26–30	71 (47.7)
31–35	44 (29.5)
> 35	17 (11.4)
Missing	0
Parity	
0	91 (61.1)
1	43 (28.9)
≥ 2	15 (10)
Missing	0
Pre-existing chronic diseases	
No	135 (90.6)
Yes	12 (8.1)
Missing	2 (1.3)
Pre-pregnancy Body Mass Index (kg/m^2^)	
Underweight (< 18.5)	8 (5.4)
Normal (18.5–24.9)	84 (56.4)
Overweight (25–29.9)	20 (13.4)
Obese (≥30)	12 (8.1)
Missing	25 (16.7)
Smoking before pregnancy	
Never	94 (63.1)
Former smoker	21 (14.1)
Missing	34 (22.8)
Alcohol consumption before pregnancy	
No	16 (10.7)
Yes	99 (66.4)
Missing	34 (22.8)
Ethnic background	
European	112 (75.2)
Non-European	4 (2.7)
Missing	33 (21.1)
Educational attainment	
Low (Basic level)	15 (10.1)
Intermediate (Secondary school)	15 (10.1)
High (Higher education)	85 (57.0)
Missing	34 (22.8)
Pre-pregnancy employment	
No	7 (4.7)
Yes	107 (71.8)
Missing	35 (23.5)
Daily hair washing	
No	97 (65.1)
Yes	18 (12.1)
Missing	34 (22.8)
Season of 2nd trimester sampling (*n*=133)	
Autumn	28 (21.2)
Winter	23 (17.3)
Spring	38 (28.6)
Summer	44 (33.1)
Missing	0
Season of 3rd trimester sampling (*n*=81)	
Autumn	23 (28.4)
Winter	21 (25.9)
Spring	12 (14.8)
Summer	25 (30.9)
Missing	0

Note: pre-existing chronic diseases include diabetes, asthma, cardiovascular diseases

Residential characteristics are described in Table 2. Study participants lived in cities (38%), towns and suburbs (62%) in Flanders, none of the participants lived in a rural area. The mean ADI of our study population was 16.4% (95% CI: 14.6, 18.1) whereas the mean 2017 ADI for the study region Antwerp was 17.6% (Statistics Flanders, n.d.). We tested the significance of the association between ADI as an area-level SES indicator and maternal educational attainment as a personal SES-indicator. We did not observe a significant association between neighborhood SES and personal SES (Spearman rank *r* = − 0.074, *p* = 0.404). A small neighborhood greenspace was accessible for 94% of participants, 76% had residential access to a large greenspace. Three-month geometric mean PM_2.5_ was 11.61 (95% CI: 11.06, 12.21) μg/m^3^ and 11.55 (95% CI: 10.95, 12.18) μg/m^3^ for 2nd trimester and 3rd trimester sampling respectively. Geometric mean NO_2_ concentrations 3 months before sampling was 23.03 (95% CI: 21.67, 24.47) μg/m^3^ for the 2nd trimester and 23.19 (95% CI: 21.5, 24.98) μg/m^3^ for the 3rd trimester, 3-month geometric mean BC concentration 1.13 (95% CI: 1.05, 1.21) μg/m^3^ for the 2nd trimester and 1.17 (95% CI: 1.07, 1.28) μg/m^3^ for the 3rd trimester. Noise exposure was covered by the Flemish strategic noise map for 144 participants, 31.5% of participating mothers was exposed to noise levels ≥ 53 dB. Geometric mean 2nd trimester HCC was 3.94 (95% CI: 3.49, 4.45) pg/mg hair, geometric mean 3rd trimester HCC was 6.12 (95% CI: 4.96, 7.56) pg/mg hair. The coefficient of variance (CV) of 2nd trim HCC was 51.5%, CV of 3rd trimester HCC was 51.8%. Second and third trimester cortisol concentrations of participants that donated a hair sample twice were moderately correlated (*n* = 65, *p* < 0.01, Pearson’s *r* = 0.571).

**Table 2:** Residential characteristics of the study participants

Variable	
**Categorical variables (*n*=149)**	*n* (%)
Neighborhood greenspace	
Access to small neighborhood greenspace	
No	9 (6.0)
Yes	140 (94.0)
Access to large neighborhood greenspace	
No	36 (24.2)
Yes	113 (75.8)
Eurostat urbanization	
Cities	56 (37.6)
Towns and suburbs	93 (62.4)
Rural	0
Noise levels	
L_den_ < 53 dB	97 (65.1)
L_den_ ≥ 53 dB	47 (31.5)
Missing	5 (3.4)
**Continuous variables**	**Geometric mean (95% CI)**
Distance to major roads (m) (*n*=149)	290 (240, 349)
2nd trimester air pollution (μg/m^3^) (*n*=133)	
NO_2 _90 days prior to sampling	23.03 (21.67, 24.47)
NO_2_ 1 year prior to sampling	24.55 (23.39, 25.76)
PM_2.5 _90 days prior to sampling	11.61 (11.06, 12.21)
PM_2.5_ 1 year prior to sampling	13.09 (12.81, 13.37)
BC 90 days prior to sampling	1.13 (1.05, 1.21)
BC 1 year prior to sampling	1.29 (1.24, 1.36)
3rd trimester air pollution (μg/m^3^) (*n*=78)	
NO_2 _90 days prior to sampling	23.19 (21.53, 24.98)
NO_2_ 1 year prior to sampling	24.70 (23.27, 26.23)
PM_2.5 _90 days prior to sampling	11.55 (10.95, 12.18)
PM_2.5 _1 year prior to sampling	12.70 (12.42, 12.99)
BC 90 days prior to sampling	1.17 (1.07, 1.28)
BC 1 year prior to sampling	1.29 (1.21, 1.37)
Ambient temperature (°Celsius)	
90 days prior to 2nd trimester sampling (*n*=133)	10.5 (9.7, 11.4)
90 days prior to 3rd trimester sampling (*n*=81)	12.6 (11.7, 13.3)
Area deprivation index (%) (*n*=149)	16.4 (14.6, 18.1)

Note: Categorical data is described as frequencies (%), continuous data is described by geometric mean with 95% confidence interval (95% CI). All data is based on the maternal residential address. Major roads include E- or N-roads. Access to small neighborhood greenspace is defined as access to > 0.2 ha (ha) of greenspace within a travel distance of 400 m (m) from residence, access to large neighborhood greenspace is defined as access to > 10 ha of greenspace within a travel distance of 800 m from residence. L_den,_ day–evening–night noise level_;_ NO_2_, nitrogen dioxide; PM_2.5_, fine particulate matter with an aerodynamic diameter ≤ 2.5 μm; BC, black carbon

Spearman rank correlations of residential exposure characteristics are presented in Table 3 (2nd trimester study population) and Table 4 (3rd trimester study population)**.**

**Table 3:** Spearman rank correlations between residential exposures in the 2nd trimester study population (n = 133)

	BC	NO_2_	Distance to major road	Small greenspace	Large greenspace	Noise	ADI
PM2.5	0.68^*^	0.68^*^	−0.15	−0.02	−0.04	0.08	0.15
	BC	0.89^*^	−0.29^*^	−0.09	−0.14	0.28^*^	0.38^*^
		NO_2_	−0.37^*^	−0.08	−0.12	0.31^*^	0.46^*^
			Distance to major road	0.01	0.17	−0.32^*^	−0.20^*^
				Small greenspace	0.43^*^	−0.14	− 0.11
					Large greenspace	−0.20^*^	−0.18^*^
						Noise	0.21

^*^Significant correlations (*p* < 0.05)

Note: Air pollutants were modelled at the maternal home address, 3-month mean concentrations were calculated. Major roads include E- or N-roads. Access to small neighborhood greenspace is defined as access to > 0.2 ha (ha) of greenspace within a travel distance of 400 m (m) from residence, access to large neighborhood greenspace is defined as access to > 10 ha of greenspace within a travel distance of 800 m from residence. Noise exposure is evaluated as exposure above the WHO health-based guideline of 53 dB Lden (day–evening–night noise level). *ADI* Area deprivation index, *NO*_*2*_ Nitrogen dioxide, *PM*_*2.5*_ Fine particulate matter with an aerodynamic diameter ≤ 2.5 μm, *BC* Black carbon

**Table 4:** Spearman rank correlations between residential exposure in the 3rd trimester study population (n = 81)

	BC	NO_2_	Distance to major road	Small greenspace	Large greenspace	Noise	ADI
PM2.5	0.61^*^	0.55^*^	−0.06	−0.19^*^	− 0.31^*^	0.01	0.04
	BC	0.89^*^	−0.24^*^	−0.22^*^	− 0.30^*^	0.18	0.31^*^
		NO_2_	−0.32^*^	−0.21^*^	− 0.28^*^	0.24^*^	0.39^*^
			Distance to major road	0.13	0.26^*^	−0.31^*^	− 0.25^*^
				Small greenspace	0.41^*^	−0.37^*^	−0.08
					Large greenspace	−0.15^*^	−0.19
						Noise	0.25^*^

^*^Significant correlations (*p* < 0.05)

Note: Air pollutants were modelled at the maternal home address, 3-month mean concentrations were calculated. Major roads include E- or N-roads. Access to small neighborhood greenspace is defined as access to > 0.2 ha (ha) of greenspace within a travel distance of 400 m (m) from residence, access to large neighborhood greenspace is defined as access to > 10 ha of greenspace within a travel distance of 800 m from residence. Noise exposure is evaluated as exposure above the WHO health-based guideline of 53 dB Lden (day–evening–night noise level). *ADI* Area deprivation index, *NO*_*2*_ Nitrogen dioxide, *PM*_*2.5*_ Fine particulate matter with an aerodynamic diameter ≤ 2.5 μm, *BC* Black carbon

We observed strong positive correlations between 3-month mean air pollutant concentrations (*r* ranged from 0.61 to 0.89). Distance to major roads was negatively correlated with NO_2_ and BC concentrations (*r* ranged from − 0.24 and − 0.37), but not with PM_2.5_ concentrations.

Access to a neighborhood greenspace did not significantly correlate with air pollutants and distance to major roads in the 2nd trimester. In the 3rd trimester study population, we did find weak negative correlations between access to a large neighborhood greenspace and air pollutants (*r* ranged from − 0.28 to − 0.31) and a weak positive association of access to a large neighborhood greenspace with distance to major road (*r* = 0.26). The ADI was weakly positively correlated with NO_2_, BC and noise exposure above the WHO guideline (*r* ranged from 0.21 to 0.46) and negatively correlated with distance to major roads and access to a large neighborhood greenspace (*r* ranged from − 0.18 to − 0.25).

In the ANOVA, season of sampling and daily hair washing were identified as significant covariates of 2nd trimester HCC, no significant covariates were identified for 3rd trimester HCC (see Table S1 for details). None of the participants reported the systemic use of glucocorticoids. Residential noise exposure above the WHO guideline (≥53 dB L_den_) was not significantly associated with 2nd or 3rd trimester HCC (*p* = 0.871, *p* = 0.190 respectively). Nor did we find significant associations between the ADI and 2nd or 3rd trimester HCC (*p* = 0.661, *p* = 0.388 resp.).

Results of the associations between air pollution exposure, access to neighborhood greenspace and maternal biological stress are presented in Table 5. We found a significant negative association between 3-month mean PM_2.5_ concentrations and 2nd trimester HCC in the unadjusted model (β = 0.81 (95% CI: 0.70, 0.95), *p* = 0.009), the association did not remain significant after adjustment for season of sampling (β = 0.87 (95% CI: 0.70, 1.08), *p* = 0.200). In the 3rd trimester, 3-month mean PM_2.5_ concentrations were not significantly associated with HCC in the unadjusted model (β = 1.20 (95%CI: 0.89, 1.62), *p* = 0.227), we observed a tendency towards a significant positive association after adjustment for season of sampling (β = 1.46 (95% CI: 1.01, 2.11), *p* = 0.051).

**Table 5:** Associations between residential exposures and hair cortisol concentrations in the second and third pregnancy trimester

	Model I		Model II	
	***p-***value	β (95% CI)	***p***-value	β (95% CI)
**Second trimester (*n*** **= 133)**				
**Exposure (p25-p75)**				
3-month mean PM_2.5_ (9.66–13.64 μg/m^3^)	**0.009**	**0.81 (0.70, 0.95)**	0.200	0.87 (0.70, 1.08)
3-month mean NO_2_ (18.37–30.22 μg/m^3^)	0.934	0.99 (0.83, 1.18)	0.287	1.10 (0.92, 1.34)
3-month mean BC (0.84–1.49 μg/m^3^)	0.551	0.94 (0.79, 1.13)	0.775	1.03 (0.84, 1.27)
Distance to major road (143–642 m)	**0.016**	**0.82 (0.70, 0.96)**	**0.011**	**0.82 (0.70, 0.95)**
Access to a small NHGS	0.117	0.68 (0.42, 1.10)	0.061	0.63 (0.38, 1.02)
Access to a large NHGS	0.073	0.77 (0.57, 1.03)	0.095	0.78 (0.59, 1.04)
**Third trimester (*n*** **= 81)**				
**Exposure (p25-p75)**				
3-month mean PM_2.5_ (9.59–13.27 μg/m^3^)	0.227*	1.20 (0.89, 1.62)	0.051*	1.46 (1.01, 2.11)
3-month mean NO_2_ (18.35–30.00 μg/m^3^)	0.016*	**1.42 (1.07, 1.88)**	0.008*	**1.53 (1.12, 2.09)**
3-month mean BC (0.84–1.48 μg/m^3^)	0.032*	**1.37 (1.03, 1.82)**	0.017*	**1.54 (1.08, 2.18)**
Distance to major road (114–598 m)	**0.040**	**0.74 (0.55, 0.99)**	0.055	0.75 (0.56, 1.01)
Access to a small NHGS	0.354	0.67 (0.28, 1.05)	0.169	0.53 (0.22, 1.32)
Access to a large NHGS	0.062	0.65 (0.41, 1.02)	**0.019**	**0.57 (0.36, 0.91)**

Note: Estimates (β) of the linear regression models are presented with their 95% confidence intervals (95%CI) as a factor increase in hair cortisol concentrations for a factor increase in exposure from the 25th to the 75th percentile (p75/p25). Model I is unadjusted, Model II is adjusted for season of sampling, Significant associations (*p* < 0.05) are marked in bold. *Associations evaluated for 78 participants. Air pollutants were modelled at the maternal home address, 3-month mean concentrations were calculated. Major roads include E- or N-roads. Access to small neighborhood greenspace is defined as access to > 0.2 ha (ha) of greenspace within a travel distance of 400 m (m) from residence, access to large neighborhood greenspace is defined as access to > 10 ha of greenspace within a travel distance of 800 m from residence. *CI* Confidence interval, *NO*_*2*_ Nitrogen dioxide, *PM*_*2.5*_ Fine particulate matter with an aerodynamic diameter ≤ 2.5 μm, *BC* Black carbon, *NHGS* Neighborhood greenspace. Significant associations (*p* < 0.05) are marked in bold

We did not observe significant associations between 3-month mean NO_2_ and BC concentrations and 2nd trimester HCC. We observed a significant positive association between 3-month mean NO_2_ concentrations and 3rd trimester HCC in the unadjusted model (β = 1.42 (95% CI: 1.07, 1.88), *p* = 0.016), the association remained significant after adjustment for season of sampling (*p* = 0.008). For an increase of 3-month mean residential NO_2_ concentrations with a factor 1.63 (18.35 μg/m^3^ (p25) to 30 μg/m^3^ (p75)), an increase of 3rd trimester HCC with a factor 1.53 (95% CI: 1.12, 2.09) was estimated in the model, adjusted for season of sampling. We also observed a significant positive association between 3-month mean BC concentrations and 3rd trimester HCC in the unadjusted model (β= 1.37 (95% CI: 1.03, 1.82), *p* = 0.032) and after adjustment for season of sampling (*p* = 0.017). For an increase of 3-month mean residential BC concentrations with a factor 1.76 (0.84 μg/m^3^ (p25) to 1.48 μg/m^3^ (p75)), an increase of 3rd trimester HCC with a factor 1.54 (95% CI: 1.08, 2.18) was estimated in the model, adjusted for season of sampling. The model including season of sampling and residential 3-month mean NO_2_ concentrations explained 7.7% of the variation in 3rd trimester HCC, season of sampling and 3-month mean BC concentrations explained 5.9% of the variation in 3rd trimester HCC.

Residential distance to a major road was negatively associated with second trimester HCC in the unadjusted model (β = 0.82 (95% CI: 0.70, 0.96), *p* = 0.016) and in the season-adjusted model (*p* = 0.011). For an increase in distance to major roads with a factor 4.49 (143 m (p25) to 642 m (p75)), a decrease of 2nd trimester HCC with a factor 0.82 (95% CI: 0.70, 0.95) was estimated in the model, adjusted for season of sampling. This model explained 7.8% of the variation in 2nd trimester HCC. Distance to major roads was also negatively associated with 3rd trimester HCC in the unadjusted model (*p* = 0.040), for an increase of distance to a major road with a factor 5.25 (114 m (p25) to 598 m (p75)), a decrease of 3rd trimester HCC with a factor 0.74 (95% CI: 0.55, 0.99) was estimated. Distance to a major road explained 4% of the variation in 3rd trimester HCC. Adjusting the model for season of sampling slightly attenuated the association (β = 0.75 (95% CI: 0.56, 1.01)*, p* = 0.055).

Access to a small neighborhood greenspace tended towards a significant negative association with 2nd trimester HCC (β = 0.68 (95%CI: 0.42, 1.10), *p* = 0.117 for the unadjusted model and β = 0.63 (95% CI: 0.38, 1.02, *p* = 0.061 for the season-adjusted model) and was not significantly associated with 3rd trimester HCC before or after adjustment for season of sampling (β = 0.67 (95%CI: 0.28, 1.05), *p* = 0.354 and β = 0.53 (95%CI: 0.22, 1.32), *p* = 0.169 resp.). Access to a large neighborhood greenspace tended towards a significant negative association with 2nd trimester HCC (β = 0.77 (95% CI: 0.57, 1.03), *p* = 0.073 for the unadjusted model, and β = 0.78 (95% CI: 0.59, 1.04), *p* = 0.095 for the season-adjusted model). Access to a large neighborhood greenspace also tended towards a significant negative association with 3rd trimester HCC in the unadjusted model (β = 0.65 (95% CI: 0.41, 1.02, *p* = 0.062), we observed a significant negative association after adjustment for season of sampling (β = 0.57 (0.36, 0.91), *p* = 0.019).

**Fig. 1:**
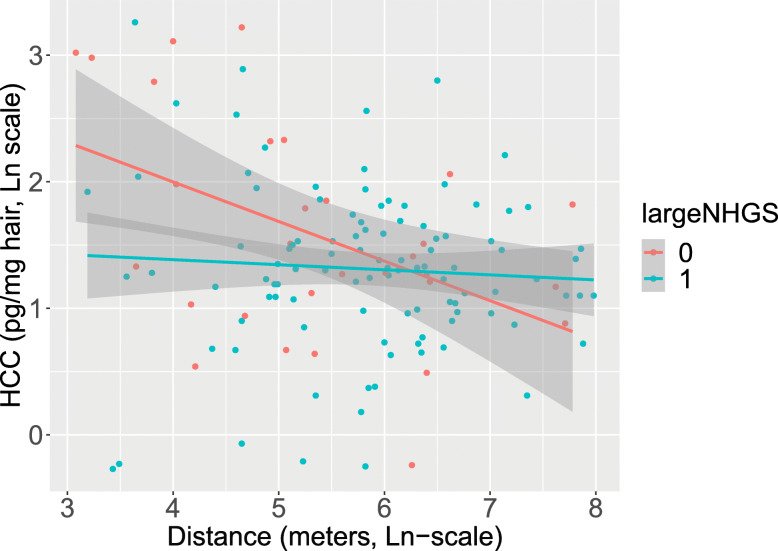
Interaction between distance to a major road and access to a large neighborhood greenspace in relation to 2nd trimester HCC

We tested whether access to a neighborhood greenspace moderated the associations between air pollution exposure, proximity to major roads and maternal biological stress. We found no significant interaction between access to a small or large neighborhood greenspace and air pollution constituents in relation to 2nd or 3rd trimester HCC (see Table S2 for details). We did observe a significant interaction between access to a large neighborhood greenspace (NHGS) and distance to major roads in relation to 2nd trimester HCC in both the unadjusted model, as presented in Fig. 1, and in the model adjusted for season of sampling (*p-interaction* = 0.021, *p-interaction* = 0.034 resp.). Distance to major roads was significantly associated with 2nd trimester HCC for participants without access to a large NHGS (β = 0.64 (95% CI: 0.48, 0.85) *p* = 0.003). The association was not significant for participants with access to a large NHGS (β = 0.92 (95% CI: 0.77, 1.11), *p* = 0.399). The season-adjusted interaction model explained 10.8% of the variations in 2nd trimester HCC.

**Fig. 2:**
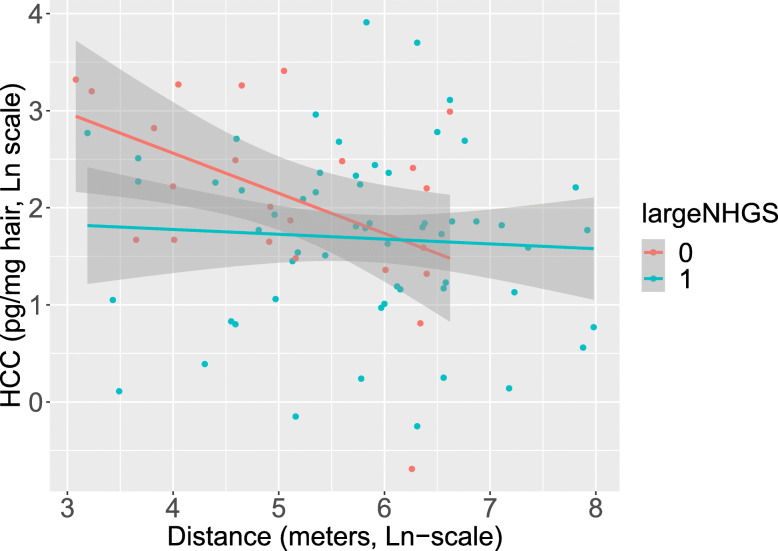
Interaction between distance to a major road and access to a large neighborhood greenspace in relation to 3rd trimester HCC

The interaction between access to a large neighborhood greenspace (NHGS) and distance to major roads in relation to 3rd trimester HCC tended towards significance in the unadjusted model (*p-interaction* = 0.073), as presented in Fig. 2, and after adjustment for season of sampling (*p*-interaction = 0.080). For pregnant women without access to a large NHGS, we found a significant association between distance to major roads and 3rd trimester HCC (β = 0.53 (95% CI: 0.30, 0.94), *p* = 0.030) after adjustment for season of sampling. The association was not significant for pregnant women with access to a large NHGS (β = 0.96 (95% CI: 0.68, 1.36), *p* = 0.816). The interaction model, adjusted for season of sampling, explained 9.6% of the variations in 3rd trimester HCC.

In a sensitivity analysis, we evaluated the significance of associations between 1-year mean PM_2.5_, NO_2_ and BC concentrations and HCC to reflect the participants’ longer-term residential exposure to air pollution. Results are presented in Table S3. Extending the exposure period did not change our results much, we found significant positive associations between 1-year mean NO_2_ concentrations and 3rd trimester HCC in the unadjusted model and in the model, adjusted for season of sampling (β = 1.49 (95% CI: 1.09, 2.04), *p* = 0.013 and β = 1.44 (95% CI: 1.05, 1.99), *p* = 0.024, resp.) and between 1-year mean BC concentrations and 3rd trimester HCC (β = 1.39 (95% CI: 1.01, 1.93), *p* = 0.046). After adjustment for season of sampling, the association between 1-year mean BC concentrations and 3rd trimester HCC slightly attenuated (*p* = 0.083, β = 1.36 (95% CI: 0.96, 1.92). The robustness of our results was also evaluated by additional adjustment of our models with daily hair washing. Our results, presented in Table S4, remained robust. Additional adjustment of the models for maternal age, pre-pregnancy BMI, educational attainment and ADI slightly strengthened the estimated associations of distance to major roads with 2nd and 3rd trimester HCC and of residential exposure to NO_2_, BC and access to large NHGS with 3rd trimester HCC (see Table S5 for details). Excluding participants of non-European origin did not change the statistical significance of the results (see Table S6 for details).

## Discussion

This study provides new insights in the relation between residential exposure to air pollution, road traffic, residential access to neighborhood greenspaces and hair cortisol as a biomarker for longer-term biological stress during pregnancy. We observed significant positive associations between residential 3-month mean NO_2_ and BC concentrations and maternal biological stress in the 3rd pregnancy trimester. It should be noted that NO_2_ exposure levels were strongly correlated with BC exposure levels (*r* = 0.89), making it impossible to disentangle the effects of both pollutants. In urban settings, road traffic is the principal source of NO_2_ and BC in ambient air [60]. We also observed a significant association between residential proximity to major roads and maternal biological stress in the 3rd pregnancy trimester in the unadjusted model, the significance slightly attenuated after adjustment of the model for season of sampling. As previously reported, residential proximity to major roads and maternal biological stress in the 2nd pregnancy trimester were significantly associated [61]. We observed a significant negative association between 3-month mean PM_2.5_ concentrations and 2nd trimester HCC in the unadjusted analysis, the association did not remain significant after adjustment for season of sampling, indicating that the unadjusted analysis may have been confounded by seasonal variations in PM_2.5_ concentrations. The difference in significant associations between traffic-related exposures and 2nd and 3rd trimester HCC may be due to the difference in study population between both trimesters or to the increase in circulating cortisol concentrations towards the end of pregnancy, which is a normal biological process [24]. Our observations are in line with recent human studies that reported associations between air pollutants and short-term variations in cortisol secretion. In a panel study among 43 students in Shanghai, residential exposure to PM_2.5_ was associated with higher serum cortisol levels [13]. In a cross-sectional analysis of 1793 adults, residential NO_2_ exposure was associated with higher wake-up salivary cortisol [14]. To our knowledge, only one epidemiological study has examined the association between personal air pollution exposure and HCC; the study, including Belgian schoolchildren and adolescents, did not find a significant relationship [62]. Pregnancy however, is a vulnerable period for both mother and fetus [63]. Several mechanisms potentially underlie the association between air pollution and biological stress during pregnancy. Air pollutants may induce oxidative stress and low grade inflammation [64]. Depending on size and chemical composition, inhaled air pollution constituents may translocate from the lungs to the systemic circulation or migrate via olfactory transport to the brain and directly interact with brain tissues including the hypothalamus [11]. During pregnancy, oxidative stress is known to be higher than in the non-pregnant state; residential exposure to air pollutants and road traffic may further amplify the level of maternal oxidative stress [65]. Oxidative stress may in turn lead to low grade inflammation and HPA axis activation, resulting in a marked increase in the secretion of cortisol into the circulation [66]. In addition to indirect activation of the HPA axis by systemic low grade inflammation, low grade inflammation in the brain may directly activate the hypothalamus [67]. Flanders, the IPANEMA study region, is characterized by a dense road network and high emissions from traffic [68]. The fraction of the Flemish population, living and working in close proximity to traffic, is high and access to neighborhood greenspace is typically limited. Interestingly, in our urban and suburban pregnancy cohort, we observed a significant negative association of access to a large neighborhood greenspace of more than 10 ha within 800 m travel distance from residence, e.g. an urban park, with 3rd trimester HCC. Moreover, access to a large neighborhood greenspace significantly moderated the association between residential proximity to major roads and maternal biological stress in the 2nd pregnancy trimester. Beneficial relationships between residential access to greenspace and hair cortisol concentrations have been described in previous studies [28, 69]; whereas other studies have reported a beneficial impact of surrounding greenness on fetal growth and birth weight [70–73]. Neighborhood greenspace may improve health by relieving psychophysiological stress, supporting physical activity, increasing social contacts and by reducing exposure to air pollution, noise and excessive heat [27, 74–76]. In our study, we found a weak inverse correlation between access to a large greenspace and residential air pollutant concentrations in the 3rd pregnancy trimester, but not in the 2nd trimester. This may suggest a moderating effect of residential access to a large greenspace, independent of the effect on air pollution exposure levels.

The added value of prospective cohort studies such as IPANEMA, is the possibility to provide more insight into early pathophysiological mechanisms, triggered by air pollution exposure, in real-world settings. The urban and suburban character of the IPANEMA cohort made it possible to go deeper into traffic-related air pollution exposure, notwithstanding the low number of participants. Residential exposure to air pollution was estimated using a high spatial resolution model, residential mobility of participants was considered. In addition to maternal traffic and air pollution exposure, we took access to neighborhood greenspaces into account. Evaluation of only one residential environmental exposure i.e. air pollution, ignoring potential interaction with other jointly occurring exposures i.e. access to greenspaces, could lead to an inaccurate estimate of the true effect of exposures [77]. We measured hair cortisol concentrations, a novel method in epidemiological studies to retrospectively determine longer-term biological stress in a non-invasive and reliable way. Hair samples were collected according to a strict protocol by trained midwives at the in-hospital consultation to avoid interindividual differences in hair collection. Some limitations of the study need to be addressed. In this study, we had a limited number of participants and did not have the same study population in the second and third pregnancy trimester, leading to differences in residential exposures. We had a considerable percentage of missing questionnaire-based data. Future prospective cohort studies, ideally including a larger number of participants from pre-conception onwards, should enhance efforts to collect questionnaires from all participants, including relevant information on time spent in residential neighborhood greenspaces, physical activity, wellbeing and health. We also recommend future studies to take the different vegetation structures of greenspaces into account and assess qualitative characteristics of greenspaces, such as amenities, aesthetics, walkability and safety. Another limitation of our study is that we assessed air pollutant concentrations by estimation, not by measurement. The exposure assessment was also limited to residential surroundings, we did not consider air pollution exposure while commuting and working. Future studies may consider additional assessment of personal air pollution exposure using portable environmental sensors [78]. IPANEMA participants were mostly of a higher socioeconomic status, it was therefore not possible to explore increased vulnerability to environmental exposure in participants with lower SES. Moreover, the neighborhood SES indicator in this study did not reflect participants with lower socio-economic status. In literature, the pattern of air pollution is often described as U-shaped, although the most deprived areas have the highest levels of poor air quality, the least deprived areas also experience higher levels of air pollutants than some other social groups [50]. In our cohort, the mean ADI was 18.1% for lower educated mothers, 15.3% for medium education mothers and, as described in literature, we observed a slightly higher ADI of 15.6% for higher educated women. Future studies should enhance efforts to include participants of all SES.

## Conclusions

This study observed significant positive associations between residential exposure to traffic-related air pollution during pregnancy and longer-term biological stress in the 2nd and 3rd trimester of pregnancy. In the 2nd trimester of pregnancy, the association was significantly moderated by residential access to a large neighborhood greenspace. Air pollution and urban spatial planning are in the center of public debate in Flanders. Because of the ubiquitous nature of traffic-related air pollution and the adverse pregnancy outcomes that have been associated with elevated maternal biological stress for both mothers and babies, even a small increase in maternal biological stress may be of public health interest. Our research, if confirmed in future studies, may provide guidance towards a more sustainable urban planning and support environmental health protection for both pregnant women and their babies.

## Supplementary Information

**Additional file 1: Table S1**. Univariate analysis of variance of possible covariates of hair cortisol concentrations. **Table S2**. Interactions of air pollutant exposure, distance to major roads with access to neighborhood greenspace in relation to hair cortisol concentrations. **Table S3**. Associations of 1-year mean residential air pollutant concentrations with hair cortisol concentrations. **Table S4**. Associations of residential exposures with hair cortisol concentrations, models additionally adjusted for daily hair washing. **Table S5**. Associations of residential exposures with hair cortisol concentrations, models additionally adjusted for age, pre-pregnancy BMI, personal and neighborhood SES. **Table S6**. Associations of residential exposures with hair cortisol concentrations, participants of non-European origin excluded

## Data Availability

The datasets used and analyzed during the current study are available from the corresponding author on reasonable request.

## Abbreviations

ADI: Area deprivation index
BC: Black carbon
BMI: Body mass index
HCC: Hair cortisol concentrations
HPA axis: Hypothalamic-pituitary-adrenal
HPLC: High-Performance Liquid Chromatography
IPANEMA: Impact of Particulate Matter on Mothers and Babies in Antwerp
IRCEL: Belgian Interregional Environment Agency
LC-MS/MS: Liquid chromatography and tandem mass spectrometry
Lden: Day–evening–night noise level
LOQ: Limit of quantification
NHGS: Neighborhood greenspace
NO_2_: Nitrogen dioxide
PM_2.5_: Particulate matter with an aerodynamic diameter ≤ 2.5 μm
RIO-IFDM: Air quality interpolation model combined with Immission Frequency Distribution Model
SES: Socioeconomic status
WHO: World Health Organization
