# Supplementary material for: Residential exposure to air pollution and access to neighborhood greenspace in relation to hair cortisol concentrations during the second and third trimester of pregnancy
Source: Environ Health. 2021 Feb 11;20:11. doi: 10.1186/s12940-021-00697-z (PMC7879652; doi:10.1186/s12940-021-00697-z)
Supplement: Supplementary file 1 — Additional file 1: Table S1. Univariate analysis of variance of possible covariates of hair cortisol concentrations. Table S2. Interactions of air pollutant exposure, distance to major roads with access to neighborhood greenspace in relation to hair cortisol concentrations. Table S3. Associations of 1-year mean residential air pollutant concentrations with hair cortisol concentrations. Table S4. Associations of residential exposures with hair cortisol concentrations, models additionally adjusted for daily hair washing. Table S5. Associations of residential exposures with hair cortisol concentrations, models additionally adjusted for age, pre-pregnancy BMI, personal and neighborhood SES. Table S6. Associations of residential exposures with hair cortisol concentrations, participants of non-European origin excluded [file 12940_2021_697_MOESM1_ESM.docx]

# Supplementary material

# Residential exposure to air pollution and access to neighborhood greenspace in relation to hair cortisol concentrations during the second and third trimester of pregnancy

Veerle Josefa Verheyen, Sylvie Remy, Nathalie Lambrechts, Eva Govarts, Ann Colles, Lien Poelmans, Els Verachtert, Wouter Lefebvre, Pieter Monsieurs, Charlotte Vanpoucke, Flemming Nielsen, Lena Van den Eeden, Yves Jacquemyn, Greet Schoeters

Table S1. Univariate analysis of variance of possible covariates of hair cortisol concentrations

Table S2. Interactions of air pollutant exposure, distance to major roads with access to neighborhood greenspace in relation to hair cortisol concentrations

Table S3. Associations of 1-year mean residential air pollutant concentrations with hair cortisol concentrations

Table S4. Associations of residential exposures with hair cortisol concentrations, models additionally adjusted for daily hair washing

Table S5. Associations of residential exposures with hair cortisol concentrations, models additionally adjusted for age, pre-pregnancy BMI, personal and neighborhood SES

Table S6. Associations of residential exposures with hair cortisol concentrations, participants of non-European origin excluded

Table S1. Univariate analysis of variance of possible covariates of hair cortisol concentrations

|  | **2nd trimester HCC** | **3rd trimester HCC** |
| --- | --- | --- |
| **Covariates of HCC** | ***p*-value** | ***p*-value** |
| Maternal age | 0.929 | 0.898 |
| Parity | 0.448 | 0.670 |
| Pre-existing diseases | 0.310 | 0.211 |
| Pre-pregnancy Body Mass Index | 0.336 | 0.267 |
| Smoking before pregnancy | 0.628 | 0.540 |
| Alcohol consumption before pregnancy | 0.776 | 0.413 |
| Educational attainment | 0.741 | 0.912 |
| Pre-pregnancy employment | 0.176 | 0.366 |
| Ethnic background | 0.106 | 0.504 |
| Area Deprivation Index | 0.661 | 0.388 |
| Residential noise exposure (L_den_) | 0.871 | 0.200 |
| Daily hair washing | 0.009 | 0.272 |
| Gestational week at sampling | 0.990 | 0.343 |
| Season of 2^nd^ trimester sampling | 0.047 | n/a |
| Season of 3^rd^ trimester sampling | n/a | 0.367 |

Note: Pre-existing chronic diseases include diabetes, asthma, cardiovascular diseases. The highest educational attainment of the mother is categorized as low (basic level)/intermediate (secondary school)/high (higher education). Ethnic background is evaluated as European/non-European. Noise exposure is evaluated as exposure above the WHO health-based guideline of 53 dB Lden (day–evening–night noise level). ADI, area deprivation index; HCC hair cortisol concentrations, n/a not applicable

Table S2. Interactions of air pollutant exposure, distance to major roads with access to neighborhood greenspace in relation to hair cortisol concentrations

| **Second pregnancy trimester** | **Model I (*n* = 133)** | **Model II (*n* = 133)** |
| --- | --- | --- |
|  |  |  |
| **Interaction** | ***p*-interaction value** | ***p*-interaction value** |
| 3-month mean PM_2.5_ x access to small NHGS | 0.428 | 0.445 |
| 3-month mean PM_2.5_ x access to large NHGS | 0.302 | 0.325 |
| 3-month mean NO_2_ x access to small NHGS | 0.905 | 0.938 |
| 3-month mean NO_2_ x access to large NHGS | 0.957 | 0.600 |
| 3-month mean BC x access to small NHGS | 0.537 | 0.759 |
| 3-month mean BC x access to large NHGS | 0.919 | 0.974 |
| Distance to major road x access to small NHGS | 0.096 | 0.120 |
| Distance to major road x access to large NHGS | **0.021** | **0.034** |
| **Third pregnancy trimester** | **Model I (*n* = 81)** | **Model II (*n* = 81)** |
|  |  |  |
| **Interaction** | ***p*-interaction value** | ***p*-interaction value** |
| 3-month mean PM_2.5_ x access to small NHGS | 0.493* | 0.348* |
| 3-month mean PM_2.5_ x access to large NHGS | 0.565* | 0.858* |
| 3-month mean NO_2_ x access to small NHGS | 0.555* | 0.341* |
| 3-month mean NO_2_ x access to large NHGS | 0.396* | 0.390* |
| 3-month mean BC x access to small NHGS | 0.552* | 0.312* |
| 3-month mean BC x access to large NHGS | 0.269* | 0.297* |
| Distance to major road x access to small NHGS | 0.200 | 0.284 |
| Distance to major road x access to large NHGS | 0.073 | 0.080 |

Note: Linear regression model I is unadjusted, model II is adjusted for season of sampling, *p*-values of the interaction terms are presented. *Results for 78 participants. All data is based on the maternal residential address. Major roads include E- or N-roads. Access to small neighborhood greenspace is defined as access to > 0.2 hectares (ha) of greenspace within a travel distance of 400 meters (m) from residence, access to large neighborhood greenspace is defined as access to > 10 ha of greenspace within a travel distance of 800 m from residence. CI, confidence interval; NHGS, neighborhood greenspace; NO_2_, nitrogen dioxide; PM_2.5_, fine particulate matter with an aerodynamic diameter ≤ 2.5 μm; BC, black carbon. Significant interactions are marked in bold.

*Table S3. Associations of 1-year mean residential air pollutant concentrations with hair cortisol concentrations*

| **Second trimester HCC (*n* = 133)** | **Model I** | | **Model II** | |
| --- | --- | --- | --- | --- |
| **Exposure (p25 - p75)** | ***p-*value** | **β coefficient (95% CI)** | ***p*-value** | **β coefficient (95% CI)** |
| 1-year mean PM_2.5_ (11.96 - 14.44 µg/m^3^) | **0.975** | 1.00 (0.83, 1.20) | 0.706 | 0.97 (0.80, 1.16) |
| 1-year mean NO_2_ (20.36 - 29.37 µg/m^3^) | 0.425 | 1.07 (0.91, 1.26) | 0.322 | 1.00 (0.92, 1.28) |
| 1-year mean BC (1.04 - 1.56 µg/m^3^) | 0.558 | 1.06 (0.88, 1.27) | 0.525 | 1.06 (0.89, 1.27) |
| **Third trimester HCC (*n* = 78)** | **Model I** | | **Model II** | |
| **Exposure (p25 - p75)** | ***p-*value** | **β coefficient (95% CI)** | ***p*-value** | **β coefficient (95% CI)** |
| 1-year mean PM_2.5_ (11.96 - 13.66 µg/m^3^) | 0.462 | 1.11 (0.84, 1.45) | 0.704 | 1.07 (0.75, 1.53) |
| 1-year mean NO_2_ (20.17 - 30.49 µg/m^3^) | **0.013** | **1.49 (1.09, 2.04)** | **0.024** | **1.44 (1.05, 1.99)** |
| 1-year mean BC (1.06 - 1.62 µg/m^3^) | **0.046** | **1.39 (1.01, 1.93)** | 0.083 | 1.36 (0.96, 1.92) |

Note: Linear regression model I is unadjusted, model II is adjusted for season of sampling. Estimates (β) are presented as a factor increase in hair cortisol concentrations for a factor increase in exposure from the 25^th^ to the 75^th^ percentile (p75/p25). All data is based on the maternal residential address. CI, confidence interval; HCC, hair cortisol concentrations; NO_2_, nitrogen dioxide; PM_2.5_, fine particulate matter with an aerodynamic diameter ≤ 2.5 μm; BC, black carbon.

Table S4. Associations of residential exposures with hair cortisol concentrations, models additionally adjusted for daily hair washing

|  | **2^nd^ trimester HCC, model adjusted for season and hair washing frequency (*n* = 103)** | |
| --- | --- | --- |
| **Exposure (p25 - p75)** | ***p*-value** | **β coefficient**  **(95% CI)** |
| 3-month mean PM_2.5_ concentrations (9.66 - 13.39 µg/m^3^) | 0.352 | 0.88 (0.68, 1.15) |
| 3-month mean NO_2_ concentrations (18.27 - 28.62 µg/m^3^) | 0.622 | 1.05 (0.85, 1.30) |
| 3-month mean BC concentrations (0.84 - 1.40 µg/m^3^) | 0.941 | 1.01 (0.80, 1.27) |
| Distance to major roads (135 - 618 m) | **0.006** | **0.77 (0.64, 0.92)** |
| Access to small NHGS | 0.074 | 0.59 (0.33, 1.05) |
| Access to large NHGS | 0.071 | 0.72 (0.51, 1.03) |
|  | **3^rd^ trimester HCC, model adjusted for season and hair washing frequency (*n* = 68)** | |
| **Exposure (p25 - p75)** | ***p*-value** | **β coefficient**  **(95% CI)** |
| 3-month mean PM_2.5_ concentrations (9.71 - 13.44 µg/m^3^) | 0.245 | 1.19 (0.88, 1.61) |
| 3-month mean NO_2_ concentrations (19.24 - 29.30 µg/m^3^) | **0.018** | **1.50 (1.07, 2.10)** |
| 3-month mean BC concentrations (0.89 - 1.50 µg/m^3^) | **0.038** | **1.48 (1.02, 2.14)** |
| Distance to major road (138 - 603 m) | 0.064 | 0.75 (0.56, 1.02) |
| Access to small NHGS | 0.144 | 0.53 (0.22, 1.25) |
| Access to large NHGS | **0.015** | **0.55 (0.34, 0.89)** |
| **Interaction second trimester** | *p*-interaction value | |
| Distance to major roads * access to large NHGS | **0.020** | |
| **Interaction third trimester** | *p*-interaction value | |
| Distance to major roads * access to large NHGS | 0.098 | |

Note: Linear regression models adjusted for season of sampling and daily hair washing. Estimates (β) are presented with their 95% confidence intervals (95%CI) as a factor increase in hair cortisol concentrations for a factor increase in exposure from the 25^th^ to the 75^th^ percentile (p75/p25). All data is based on the maternal residential address. Major roads include E- or N-roads. Access to small neighborhood greenspace is defined as access to > 0.2 hectares (ha) of greenspace within a travel distance of 400 meters (m) from residence, access to large neighborhood greenspace is defined as access to > 10 ha of greenspace within a travel distance of 800 m from residence. CI, confidence interval; HCC, hair cortisol concentrations, NHGS, neighborhood greenspace; NO_2_, nitrogen dioxide; PM_2.5_, fine particulate matter with an aerodynamic diameter ≤ 2.5 μm; BC, black carbon. Significant associations and interactions are marked in bold.

Table S5. Associations of residential exposures with hair cortisol concentrations, models additionally adjusted for age, pre-pregnancy BMI, personal and neighborhood SES

|  | **2nd trim HCC, models adjusted for season, age, pre-pregnancy BMI and SES (*n* = 102)** | |
| --- | --- | --- |
| **Exposure (p25 - p75)** | ***p*-value** | **β coefficient**  **(95% CI)** |
| 3-month mean PM_2.5_ concentrations (9.68 – 13.41 µg/m^3^) | 0.215 | 0.84 (0.63, 1.11) |
| 3-month mean NO_2_ concentrations (18.40 – 28.82 µg/m^3^) | 0.298 | 1.14 (0.89, 1.47) |
| 3-month mean BC concentrations (0.84 – 1.40 µg/m^3^) | 0.643 | 1.07 (0.81, 1.41) |
| Distance to major road (131 – 618 m) | **0.010** | **0.76 (0.62, 0.94)** |
| Access to small NHGS | 0.051 | 0.53 (0.29, 1.01) |
| Access to large NHGS | 0.124 | 0.74 (0.51, 1.09) |
|  | **3rd trim HCC, models adjusted for season, age, pre-pregnancy BMI and SES (*n* = 69)** | |
| **Exposure (p25 - p75)** | ***p*-value** | **β coefficient**  **(95% CI)** |
| 3-month mean PM_2.5_ concentrations (9.47 – 13.33 µg/m^3^) | 0.211 | 1.30 (0.86, 1.96) |
| 3-month mean NO_2_ concentrations (19.13 – 29.75 µg/m^3^) | **0.009** | **1.65 (1.14, 2.38)** |
| 3-month mean BC concentrations (0.89 – 1.50 µg/m^3^) | **0.016** | **1.64 (1.10, 2.45)** |
| Distance to major road (105 – 603 m) | **0.038** | **0.72 (0.53, 0.98)** |
| Access to small NHGS | 0.091 | 0.46 (0.19, 1.14) |
| Access to large NHGS | **0.004** | **0.47 (0.28, 0.77)** |
| **Interaction second trimester** | *p*-interaction value | |
| Distance to major roads * access to large NHGS | **0.025** | |
| **Interaction third trimester** | *p*-interaction value | |
| Distance to major roads * access to large NHGS | 0.180 | |

Note: Linear regression modes adjusted for season of sampling, maternal age, pre-pregnancy BMI ≥ 25, personal SES (educational attainment) and neighborhood SES (ADI > 16.4%). Estimates (β) are presented with their 95% confidence intervals (95%CI) as a factor increase in hair cortisol concentrations for a factor increase in exposure from the 25^th^ to the 75^th^ percentile (p75/p25). All data is based on the maternal residential address. Major roads include E- or N-roads. Access to small neighborhood greenspace (NHGS) is defined as access to > 0.2 hectares (ha) of greenspace within a travel distance of 400 meters (m) from residence, access to large neighborhood greenspace (NHGS) is defined as access to > 10 ha of greenspace within a travel distance of 800 m from residence. CI, confidence interval; HCC, hair cortisol concentrations; NHGS, neighborhood greenspace; NO_2_, nitrogen dioxide; PM_2.5_, fine particulate matter with an aerodynamic diameter ≤ 2.5 μm; BC, black carbon, ADI area deprivation index, BMI body mass index. Significant associations and interactions are marked in bold.

Table S6. Associations of residential exposures with hair cortisol concentrations, participants of non-European origin excluded

|  | **2nd trimester HCC, model adjusted for season of sampling (*n* = 102)** | |
| --- | --- | --- |
| **Exposure (p25-p75)** | ***p*-value** | **β coefficient**  **(95% CI)** |
| 3-month mean PM_2.5_ concentrations (9.66 – 13.42 µg/m^3^) | 0.091 | 0.80 (0.62, 1.04) |
| 3-month mean NO_2_ concentrations (18.12 – 28.11 µg/m^3^) | 0.919 | 1.03 (0.81, 1.26) |
| 3-month mean BC concentrations (0.83 – 1.41 µg/m^3^) | 0.690 | 0.95 (0.75, 1.21) |
| Distance to major road (136 – 635 m) | **0.009** | **0.77 (0.64, 0.94)** |
| Access to small NHGS | 0.065 | 0.57 (0.33, 1.04) |
| Access to large NHGS | 0.231 | 0.80 (0.56, 1.15) |
|  | **3rd trimester HCC, model adjusted for season of sampling (*n* = 68)** | |
| **Exposure (p25 – p75)** | ***p*-value** | **β coefficient**  **(95% CI)** |
| 3-month mean PM_2.5_ concentrations (9.29 – 12.98 µg/m^3^) | 0.219 | 1.27 (0.86, 1.87) |
| 3-month mean NO_2_ concentrations (17.89 – 28.11 µg/m^3^) | **0.020** | **1.53 (1.07, 2.18)** |
| 3-month mean BC concentrations (0.82 – 1.39 µg/m^3^) | **0.036** | **1.53 (1.03, 2.27)** |
| Distance to major road (138 – 651m) | 0.075 | 0.76 (0.56, 1.03) |
| Access to small NHGS | 0.083 | 0.47 (0.20, 1.11) |
| Access to large NHGS | **0.046** | **0.61 (0.38, 0.99)** |
| **Interaction model second trimester** | *p*-interaction value | |
| Distance to major roads * access to large NHGS | **0.036** | |
| **Interaction model third trimester** | *p*-interaction value | |
| Distance to major roads * access to large NHGS | 0.145 | |

Note: Linear regression modes adjusted for season of sampling. Estimates (β) are presented with their 95% confidence intervals (95%CI) as a factor increase in hair cortisol concentrations for a factor increase in exposure from the 25th to the 75th percentile (p75/p25). All data is based on the maternal residential address. Major roads include E- or N-roads. Access to small neighborhood greenspace (NHGS) is defined as access to > 0.2 hectares (ha) of greenspace within a travel distance of 400 meters (m) from residence, access to large neighborhood greenspace (NHGS) is defined as access to > 10 ha of greenspace within a travel distance of 800 m from residence. CI, confidence interval; HCC, hair cortisol concentrations; NHGS, neighborhood greenspace; NO_2_, nitrogen dioxide; PM_2.5_, fine particulate matter with an aerodynamic diameter ≤ 2.5 μm; BC, black carbon, ADI area deprivation index, BMI body mass index. Significant associations and interactions are marked in bold.
